# Changes of Functional and Directed Resting-State Connectivity Are Associated with Neuronal Oscillations, ApoE Genotype and Amyloid Deposition in Mild Cognitive Impairment

**DOI:** 10.3389/fnagi.2017.00304

**Published:** 2017-09-20

**Authors:** Lars Michels, Muthuraman Muthuraman, Abdul R. Anwar, Spyros Kollias, Sandra E. Leh, Florian Riese, Paul G. Unschuld, Michael Siniatchkin, Anton F. Gietl, Christoph Hock

**Affiliations:** ^1^Clinic of Neuroradiology, University Hospital of Zurich Zurich, Switzerland; ^2^MR-Center, University Children’s Hospital Zurich Zurich, Switzerland; ^3^Clinic for Neurology, University of Kiel Kiel, Germany; ^4^Clinic for Neurology, University of Mainz Mainz, Germany; ^5^Division of Psychiatry Research and Psychogeriatric Medicine, University of Zurich Zurich, Switzerland; ^6^Institute of Medical Psychology and Medical Sociology, Christian-Albrechts-University of Kiel Kiel, Germany

**Keywords:** mild cognitive impairment, EEG, directed connectivity, amyloid deposition, apolipoprotein ε4

## Abstract

The assessment of effects associated with cognitive impairment using electroencephalography (EEG) power mapping allows the visualization of frequency-band specific local changes in oscillatory activity. In contrast, measures of coherence and dynamic source synchronization allow for the study of functional and effective connectivity, respectively. Yet, these measures have rarely been assessed in parallel in the context of mild cognitive impairment (MCI) and furthermore it has not been examined if they are related to risk factors of Alzheimer’s disease (AD) such as amyloid deposition and apolipoprotein ε4 (ApoE) allele occurrence. Here, we investigated functional and directed connectivities with Renormalized Partial Directed Coherence (RPDC) in 17 healthy controls (HC) and 17 participants with MCI. Participants underwent ApoE-genotyping and Pittsburgh compound B positron emission tomography (PiB-PET) to assess amyloid deposition. We observed lower spectral source power in MCI in the alpha and beta bands. Coherence was stronger in HC than MCI across different neuronal sources in the delta, theta, alpha, beta and gamma bands. The directed coherence analysis indicated lower information flow between fronto-temporal (including the hippocampus) sources and unidirectional connectivity in MCI. In MCI, alpha and beta RPDC showed an inverse correlation to age and gender; global amyloid deposition was inversely correlated to alpha coherence, RPDC and beta and gamma coherence. Furthermore, the ApoE status was negatively correlated to alpha coherence and RPDC, beta RPDC and gamma coherence. A classification analysis of cognitive state revealed the highest accuracy using EEG power, coherence and RPDC as input. For this small but statistically robust (Bayesian power analyses) sample, our results suggest that resting EEG related functional and directed connectivities are sensitive to the cognitive state and are linked to ApoE and amyloid burden.

## Introduction

Mild cognitive impairment (MCI) is a syndrome, identified by formal testing, which is characterized by single or multiple cognitive complaints (Albert et al., 2011). The occurrence of MCI is accompanied on the neuronal level by structural and functional alterations, such as gray matter atrophy (Convit et al., 1997; Jack et al., 1999), reduced cerebral blood flow (Hirao et al., 2005; Chen et al., 2011), or altered neuronal oscillations. The latter is seen as abnormalities of power density at specific frequency bands in quantitative electroencephalography (qEEG; Huang et al., 2000; Jelic et al., 2000; Hatz et al., 2013). Hippocampal and temporo-parietal atrophy is linked to qEEG markers in MCI (Moretti et al., 2007, 2008, 2009b, 2013).

MCI can be due to underlying Alzheimer’s pathology and MCI due to Alzheimer’s disease (AD) is separated from Alzheimer’s dementia by the grade of functional impairment. The characteristic pathological hallmarks of AD are extracellular accumulation of amyloid-beta (Aβ) in the so-called amyloid-plaques and intraneuronal deposits of hyperphosphorylated tau peptides. Elevated amyloid deposition can trigger aberrant patterns of structural integrity in cognitively normal elderly individuals (Becker et al., 2011; Schreiner et al., 2014) and also changes neuronal circuit activity at the network level (Sperling et al., 2009; Vannini et al., 2012; Steininger et al., 2014). Disturbed neuronal circuit activity is reflected by abnormal mechanisms of cortical neural synchronization and coupling that generate resting state EEG rhythms. Previous studies in MCI or AD have shown disrupted functional EEG coherence (i.e., coupling), between electrode pairs (Jiang, 2005; Tao and Tian, 2005; Jiang and Zheng, 2006; Zheng et al., 2007) and altered effective connectivity (i.e., causal effect of one electrode over another). Reduced parietal-to-frontal directional information flow has been reported in amnestic MCI and mild AD (Babiloni et al., 2010). This is in line with the idea of a common pathophysiological background for MCI and AD (Vecchio and Babiloni, 2011). Using qEEG and statistical pattern recognition method, it was recently shown that qEEG can separate patients with AD from healthy elderly individuals with a sensitivity of 84% and a specificity of 81% (Engedal et al., 2015). Other EEG studies showed a loss of long-range EEG synchronicity between fronto-parietal and fronto-temporal electrode pairs in MCI and AD patients (Dauwels et al., 2009, 2010).

Homozygosity for the apolipoprotein ε4 allele (ApoE4/E4) is considered the strongest genetic risk factor for developing sporadic AD. Apart from atrophy of medial temporal structures, ApoE can be considered as possible and predictive (bio-)marker for early AD (Elias-Sonnenschein et al., 2011). Using qEEG, an improved differentiation rate of patients with suspected AD was demonstrated when ApoE status was added as classification parameter (Hatz et al., 2013).

Despite the important role of amyloid deposition and ApoE in neurodegeneration and the interrelation between them both (as ApoE-genotype is the strongest risk factor for high amyloid-deposition in healthy subjects), there is no study which has examined the influence of cortical amyloid deposition—together with ApoE—on EEG parameters in MCI. The present study aims to examine the link between amyloid deposition and ApoE and EEG measures such as power but also functional coherence and effective connectivity. The advantage is the ability to test any dependence of amyloid deposition and ApoE on functional network properties, solely based on non-invasive and resting-state EEG recordings. Recently, we showed that renormalized partial directed coherence (RPDC) tracks directed connectivity differences between children and adults in subcortical and cortical areas (Michels et al., 2013). Based on this finding, we hypothesize that RPDC is suitable to identify aging-related causal effects on the brain surface and subcortical brain structures such as the hippocampus, i.e., structures that are functionally and structurally impaired in MCI and AD. We applied a support vector machine (SVM) analysis to identify the strongest influence of examined parameters on the classification between MCI and cognitively unaffected volunteers.

## Materials and Methods

### Participants

We studied 17 right-handed participants with (amnestic) MCI diagnosed according to standard criteria and 17 right-handed healthy controls (HC). Groups did not differ in age, sex, or level of education (Table 1). All volunteers were recruited from longitudinal cohort studies (Gietl et al., 2015; Riese et al., 2015). They underwent a comprehensive clinical work-up including investigation of neurological status, Mini-Mental state examination (MMSE, see Table 1), and neuropsychological parameters. For the latter, tests from the Consortium to Establish a Registry for AD (CERAD)-plus battery (Morris et al., 1988, 1989) were applied including tests on verbal and non-verbal memory (raw values were z-transformed for further statistical analysis). None of the participants were diagnosed with AD, epilepsy or stroke, but one had diabetes. None were smokers (<2 cigarettes/day), drinkers (<2 drinks/week), or depressive as assessed using the Hamilton rating scale questionnaire (HAMILTON-D score <13, i.e., not present to mild depression; Hamilton, 1960). There were no other neurological or relevant general medical symptoms or disorders. Participants gave written informed consent prior to study participation (and were not paid for their participation). Ethics approval was given by the cantonal ethics commission (KEK) Zurich prior to the study. All procedures performed in studies involving human participants were in accordance with the ethical standards of the institutional and/or national research committee and with the 1964 Helsinki declaration and its later amendments or comparable ethical standards.

**Table 1:** Demographics.

Group	Size	Age Mean (SD)	Sex (M = male F = female)	Aβ positive	Global Aβ load	ApoE4 carriers	MMSE (0–30)	CERAD recall (*z*-value)	Education (years)
HC	17	71.8 (4.6)	13 M/5 F	5	1.24 (0.2)	4	29.65 (0.6)	0.75 (1.2)	15.35 (2.7)
MCI	17	72.1 (4.6)	13 M/5 F	6	1.45 (0.2)	5	28.41 (0.5)	−0.95 (1.1)	14.76 (2.9)
Statistic (*t*-test or χ^2^)	*n.s*.	*0.107*	*n.s*.	*n.s*.	*0.070*	*n.s*.	*0.002*	*0.00005*	*0.656*

### Genotyping

ApoE genotyping was performed as previously described (Hixson and Vernier, 1990). Participants were classified according to their ApoE4-status. In the MCI group we had two APOE4-negative (i.e., without ε4 allele occurrence) subgroups: E2/E3 (one participant) and E3/E3 (11 participants), and two ApoE4-positive subgroups: E3/E4 (four participants) and E4/E4 (one participant).

### Positron Emission Tomography (PET) Acquisition

The positron emission tomography (PET) acquisition procedure has been previously published (Steininger et al., 2014; Gietl et al., 2015). PET scanning was performed at the PET Center of the Division of Nuclear Medicine, Zürich University Hospital on GE Discovery Scanners. Each participant received an antecubital venous injection of approximately 350 MBq of 11-C-PiB (PIB from now on). A 70-min dynamic PET scan (4 × 15, 8 × 30, 9 × 60, 2 × 180 and 10 × 300 s) was performed. In subjects not being able to lie still in the scanner for this time period a static image (50–70 min post injection) was acquired for estimation of PiB-binding. Voxel spacing was 2.34 × 2.34 × 3.27 mm. All image processing was done automatically under visual control with PMOD PNEURO tool Version 3.4 (PMOD LTD, Zurich, Switzerland). PET was co-registered to the individual MR. Segmentation was performed on the individual MRI and volume of interest (VOI) boundaries were defined by at least 50% gray matter probability. A maximum probability atlas (Hammers N30R83; Hammers et al., 2003; Gousias et al., 2008) was used to define these VOIs based on the individual segmentation of gray and white matter. For the final statistics, the MR was normalized to MNI space and the combined transformation matrices PET to MR and MR to MNI were applied to the PET data. For calculation of cortical *standard uptake value ratio* (SUVR), average PiB-uptake frames 50–70 min in all bilateral cortical brain structures of the Hammers N30R83 atlas^1^ including allocortical hippocampus but excluding occipital lobe, insula, primary motor and sensorimotor cortices—were merged using a volume-weighted averaging procedure, ensuring that larger VOIs contribute more to the combined signal that smaller VOIs. The average uptake in this merged region divided by the average bilateral cerebellar gray matter uptake (cerebellar reference) gave the cortical PiB-SUVR. The cortical SUVR cut-off for defining a subject as amyloid-positive (PiB+) was derived from 93 healthy volunteers and was ≥1.265 as described before (Vandenberghe et al., 2010).

### EEG Recording

The EEG was recorded during resting state (eyes closed, 5 min) with 64 sintered Ag/AgCl electrodes using the “BrainCap” (Falk-Minow Services, Herrsching-Breitbrunn, Germany). Electrode impedance was kept below 10 kΩ (after subtraction of the value of the safety resistors). EEG montage was based on a selection of 10–20 system positions (Brem et al., 2010). Specifically, the following 60 scalp electrodes were used: Fp1, Fp2, F3, F4, C3, C4, P3, P4, O1, O2, F7, F8, T7, T8, P7, P8, Iz, Cz, Pz, FC1, FC2, CP1, CP2, FC5, FC6, CP5, CP6, TP9, TP10, Fpz, Oz, FT9, FT10, PO9, PO10, C1, C2, PO1, PO2, Fz, AFz, F5, F6, FT7, FT8, FC3, FC4, C5, C6, TP7, TP8, CP3, CP4, P5, P6, OI1, OI2, FCz, CPz and POz. F1 served as recording reference and F2 was the ground electrode. Data were transmitted to two high-input impedance amplifiers (BrainVision MR+, Brain Products Co., Gilching, Germany; 250 Hz low-pass filter, 10 s time constant, 16-bit resolution, 32 mV dynamic range), which were connected to the EEG recording computer via fiber optic cables. Vigilance was briefly checked in the middle of the recording by the simple question: “Are you still awake?”.

### EEG Analysis

EEG preprocessing and analysis was performed using Brain Vision Analyzer 1.05. Muscle artifacts were first removed using manual data inspection by an experience neurophysiologist. In addition, we removed the part of the EEG during which we checked the vigilance status and any EEG section showing signs of sleep or drowsiness. EEG was digitally bandpass-filtered (0.5–70 Hz, 24 dB/oct and 50 Hz Notch) and downsampled to 256 Hz. An infomax independent component analysis (ICA; Delorme and Makeig, 2004) was then applied and ICA components were profiled by their topography, activation time course and spectrogram. Components clearly assigned to movement, e.g., (rarely occurring) eye blinks (Jung et al., 2000) were excluded from the back projection. EEG from all scalp channels were then transformed to the average reference (Lehmann and Skrandies, 1980), and EEG segments with remaining artifacts were removed. For all groups, the mean overall data length after these pre-processing steps did not differ between groups (HC: 4.2 min ± 0.2 min, MCI: 4.1 min ± 0.3 min, *p* > 0.05, two-tailed paired *t*-test). Next, the power spectral density was estimated, whereby the EEG signal was parsed in 2 s windows. For each of these segments a fast Fourier transformation (FFT, Hanning window: 10%, frequency resolution of 0.25 Hz) was computed per electrode and averaged across segments. The average band power was calculated as the integrated area under the absolute power spectrum in the specific frequency band of interest, divided by the width (in points) of the specific frequency band. Spectral band source mean power and synchronization analysis (see below) across all sources were examined for delta (1–3 Hz), theta (4–7 Hz), alpha (8–13 Hz), beta (14–30 Hz) and gamma (30–49 Hz).

### Analysis of Coherent Sources

A full description of the coherence analysis is given elsewhere (Michels et al., 2013). The total interaction strength, which is the mean coherence across all the sources for a particular frequency band during the eyes closed condition (from now on called coherence), was analyzed using a beamforming approach called Dynamic Imaging of Coherent Sources (DICS; Sekihara and Scholz, 1996; Gross et al., 2001; Hillebrand and Barnes, 2005). There are two major constraints in this analysis: first, the analysis is created on a single dipole model, which is not linearly correlated to other dipoles and second, the signal-to-noise ratio is sufficiently high (Gross et al., 2001). A fixed dipole model was used, in which the dipole source, which is responsible for the measured EEG potentials during an epoch, remains at a constant location. Furthermore, the dipole moment vector maintains a constant orientation throughout the epoch and only the magnitude varies. To determine coherence between brain areas, the spatial maximum of the power at respective frequency bands was identified from the grand average power maps across all the subjects and then defined as the seed region based on the assumption that the coherence between the reference voxel and itself is always 1. In the next step this area of the brain or the activated voxels are considered as noise in order to find further coherent areas in the brain (Schoffelen et al., 2008). In this way, we are able to identify the whole network involved in the brain for a particular frequency band oscillation. The selection of the reference region and the subsequent network sources was done on an automatic basis. The output of the beamformer at a voxel in the brain can be defined as a weighted sum of the output of all EEG channels (Van Veen et al., 1997). The weights determine: (1) the spatial filtering characteristics of the beamformer and are selected to; (2) increase the sensitivity to signals from a given voxel; and (3) additionally reduce the contribution of signals from any noise sources (at different locations). The frequency components and their linear interaction are represented as a cross-spectral density (CSD) matrix. The two measures, which can be derived from the CSD matrix, are power and coherence. Coherence can be estimated by normalizing the CSD between two signals with their power spectral densities. In order to visualize power and coherence in the brain at a given frequency range, a linear transformation is used based on a constrained optimization problem, which acts as a spatial filter (Van Veen et al., 1997). The spatial filter was applied to a large number of voxels covering the entire brain, assigning to each voxel a specific value of coherence. A voxel size of 5 mm was used in this study. The beamformer weights for a given source (at a location of interest) are determined by the data covariance matrix and the forward-solution (lead-field matrix—LFM). The LFM was estimated with specified models for the brain. In this study, the brain was modeled by a more complex, five-concentric-sphere model (de Munck and Peters, 1993; Van Uitert and Johnson, 2002) with a single sphere for each layer corresponding to the white matter, gray matter, cerebral spinal fluid, skull and skin. The volume conductor model was created using standard T1-weighted magnetic resonance images. Part of the forward modeling and the source analysis was done using the open source software FieldTrip (Oostenveld et al., 2011). For both groups, the head was modeled using the radius and the position of the sphere with the standard electrode locations, that is, the same head model was used for HC and MCI. The LFM contains the information about the geometry and the conductivity of the model. The complete description of the solution for the forward problem has been described previously elsewhere (Muthuraman et al., 2010). The brain region representing the strongest power in a specific frequency band can subsequently be used as a reference region for cortico-cortical coherence analysis. Areas were selected by a within-subject surrogate analysis to define the significance level, which was then used to identify areas as activated voxels to be considered as noise for subsequent runs of the source analysis. In order to create tomography maps, a spatial filter using a voxel size of 5 mm was applied to a large number of voxels (covering the entire brain). Once coherent brain areas were identified, their activity was extracted from the surface EEG (source space).

### Directionality Analysis

Coherence only reveals components that are mutually correlated to two signals—in the frequency domain—but does not inform about the direction of information flow between signals. In contrast, RPDC is a technique, based on the perspective of Granger causality (time domain), performed in the frequency domain to detect causal influences (i.e., directed connectivity) in multivariate stochastic systems and provides information on the direction of information flow between the sources (Schelter et al., 2009). The multivariate model was based strictly on causality (i.e., not taking into account zero-lagged or instantaneous influences) and was used to model the pooled source signal estimates by an autoregressive process to obtain the coefficients of the signals in the defined frequency band. The open source Matlab (The MathWorks, Inc., Natick, MA, USA) package ARFIT^2^ was used to estimate the autoregressive coefficients from the spatially filtered source signals (Neumaier and Schneider, 2001; Schneider and Neumaier, 2001). The correct model order required for the determination of these coefficients was estimated by minimizing the Akaike information criterion (Akaike, 1974). This criterion reflects a measure of the relative goodness of fit which has the minimum loss of information for the resulting statistical model with an optimal order (Ding et al., 2000). After estimating the RPDC values the significance level was calculated from the applied data using a bootstrapping method (Kaminski et al., 2001).

Since the information flow between brain areas is difficult to estimate from EEG measurements, due to the presence of noise and bias of volume conduction (Nolte et al., 2004b), any effective connectivity measure (here, RPDC) has to be carefully tested for its reliability to detect the underlying neuronal interactions during any functional state of interest (here, resting state). In this context, some authors used the imaginary part of coherence (Nolte et al., 2004a; Dubovik et al., 2012) or time reversal technique (TRT; Haufe et al., 2013; Michels et al., 2013). In a simulation study, Haufe et al. (2013) demonstrated that TRT is a suitable method to alleviate the influence of weak asymmetries (e.g., non-causal interactions caused by zero-lagged, instantaneous coherences (i.e., volume conduction)) on the result of any causal measure, while maintaining or even amplifying the influence of strong asymmetries (e.g., time-lagged causal interactions not caused by volume conduction). Hence, TRT was applied as a second significance test on the connections already identified by RPDC using bootstrapping as a data-driven surrogate significance test. Accordingly, the RPDC asymmetries should be insensitive to contributions from volume conduction or other instantaneous interactions. In addition, our RPDC asymmetry calculation should completely revert by applying TRT, and therefore be only sensitive to strong causal interactions. In another study, it was demonstrated that PDC is insensitive to volume conduction (Joffe, 2008). We applied TRT on the RPDC values for both groups (HC and MCI) during the 5-min eyes-closed EEG run.

### Support Vector Machine (SVM) Classification Analysis

In classification analysis, SVM is a powerful tool for nonlinear classification between two (or more) data sets (here nonlinear EEG data from HC and MCI). The algorithm searches for an optimally separating-threshold between the two data sets by maximizing the margin between classes’ closest points (Cortes and Vapnik, 1995). The points lying on the boundaries are called support vectors, and the middle of the margin is the optimal separating threshold. For the clinical parameters (PiB and ApoE), which were linear tested with fitting a Weibull distribution, we used the SVM with a linear kernel (Cortes and Vapnik, 1995). In most cases, the linear separator is not ideal for the non-clinical data based on EEG measures so a projection into a higher-dimensional space is performed where the data points effectively become linearly separable. Here, we have used the polynomial function kernel for this projection due to its good performance as discussed in Cortes and Vapnik (1995) and used the grid search (min = 1; max = 10) to find the few optimal input regularization parameters, namely C (Type of classification algorithm), which is the capacity constant. The parameter C should be carefully chosen because the larger the C, the more the error is penalized (i.e., leads to over-fitting) so we tested values in the range of 1–1000 and chose a gamma of 0.25 for the polynomial kernel function (which represents the data for the cross validation). The selection was checked by 10-fold cross validation by taking 75% of the data for training and 25% for testing. A soft-margin classifier of the calculated network topology measurements was used for every parameter, and misclassifications were weighted by a penalty constant C. In order to optimize classification accuracy this was calculated for every classifier. The validation scheme was used to assess whether the included parameters of power, coherence and (effective) connectivity allow automated classification between groups. The vectors from the HC and MCI patients that were included for the classification are source power, coherence and RPDC values from five frequency bands. The classification was conducted separately for each analyzed parameter and finally for all the parameters together. We reported the overall, training and testing accuracy. The SVM analysis was repeated with age as covariate in the model.

### Statistical Analysis

Frequency-band specific spectral source mean power differences were assessed by two-tailed *t*-tests. The significance of the sources was tested by a within-subject surrogate analysis, in which the surrogates were estimated by a Monte Carlo random permutation shuffling one-second segments within each subject 100 times. The *p*-value for each of these 100 random permutations was estimated and then the 99th percentile *p*-value was taken as the significance level in each subject (Muthuraman et al., 2012). To ensure that any reported results (which are all calculated for pre-defined frequency bands) are not confounded by group differences in individuals’ alpha frequency (IAF), we also estimated and compared individual band limits calculated as a percentage of the IAF (Doppelmayr et al., 1998), as done in our recent publication (Michels et al., 2013). In brief, the IAF was calculated from the mean of all EEG channels (excluding eye channels). According to the IAFs, the lower and upper boundaries of the other frequency bands (delta, theta and beta) were defined within 10% of the predefined band edges. For instance, if the IAF was 10.1 Hz, the lower band edge for the delta band is 1.01 Hz (0.1 (10% of 1 Hz) × 10.1 Hz) and the upper edge is 3.03 (0.3 × 10.1 Hz). Next, we estimated the median frequency band values across all participants to inspect whether those values lie in the range of the defined frequency bands, and whether the values differ within (one-sample *t*-tests) and between (paired *t*-tests) groups. To test differences in demographic variables between groups, two-sample *t*-tests were applied on MMSE scores, age, and education and χ^2^-Test on sex and ApoE (dummy coded: ApoE4+ and ApoE4−).

Next, for the statistical comparison of the power, coherence and RPDC values, the source power (first source), mean coherence (or interaction strength) and mean RPDC values between all the sources was estimated for testing the significance between HC and MCI patients. A Friedman two-way analysis of variance test was then performed to test for significant differences between all these values.

In addition, we tested for significant group differences by comparing (bidirectional) RPDC from one identified source to all sources, in order to identify mean information flow differences from one source to all sources between HC and MCI. For all statistical analyses, the significance level was kept at *p* < 0.05 and results were corrected for multiple comparisons using Bonferroni correction.

Due to the non-normal distribution of Aβ (*p* < 0.001, Kolmogorov-Smirnov test), and the ordinal character of ApoE (MCI: ε2/ε3, ε3/ε3, ε3/ε4, ε4/ε4), we applied nonparametric Spearman’s rank order correlation analysis to examine its link to (source) power, coherence, and RPDC in both HC and MCI. However, we did not perform this analysis for ApoE in HC, as ApoE was dichotomous (ε3/ε3, ε3/ε4). In addition, we computed partial correlations between EEG parameters and PIB (and ApoE) with age as nuisance variable in the analysis. Because of massive multiple testing (3 (EEG measures: power, coherence, RPDC) × 5 (frequency bands) × 2 (amyloid deposition and ApoE status) −>30 tests), we corrected for multiple comparisons using bootstrapping (with 1000 iterations, simple sampling) to achieve *p* < 0.05 (corrected). The 95% confidence interval (*CI*) is reported as well. We performed Pearson correlation analyses between EEG measures and cognitive test scores (e.g., CERAD or VLMT). All results were adjusted for multiplicity (for each EEG frequency band: 3 EEG measures (power, coherence, RPDC) × 12 cognitive measures = 36 tests) to minimize inflation of type I error using a less conservative method (i.e., bootstrapping with 1000 samples, 95% CI) compared to Bonferroni correction. All statistical analyses were performed in SPSS V22.

### Bayesian Power Analyses

We also tested if the examined sample size was large enough to call any of the observed effect size (i.e., classification accuracy) reliable. To test this, we applied Bayesian power analyses using the freely available software Bayesian estimation (BEST; Kruschke, 2013, 2014). The parameters which showed ≥75% in the SVM classifying both groups were used as inputs *y*_1_ (HC) and *y*_2_ (MCI) for the Markov-Chain-Monte-Carlo simulation with 100,000 sampling steps. We estimated five outputs for each input parameter namely the mean, standard deviation, posterior distribution prediction, normality and effect size.

## Results

### Demographics

The two groups (HC and MCI) did not differ in age, sex and education (Table 1, all *p* > 0.1). Also, age did not differ between MCI PiB+ and MCI PiB− individuals (*p* = 0.53, *t* = 1.1, unpaired *t*-test) or between all PiB+ and PiB− individuals (*p* = 0.12, *t* = 1.7, unpaired *t*-test). The mean MMSE scores were significantly higher (*p* = 0.0012, *t* = 3.4, unpaired *t*-test) for HC (29.6, SD: 0.6) than for MCI (mean: 28.4, SD: 1.4). Also, CERAD recall scores were lower for MCI than HC (Table 1).

### PiB-PET

Amyloid deposition was higher (trend) in the group of MCI subjects than in the HC group (see Table 1, *p* = 0.07, *t* = 1.9, unpaired *t*-test). Only in the MCI group, PiB correlated to ApoE (*r* = 0.55, *p* = 0.02).

### EEG: Undirected Source Coherence

**Figure 1:**
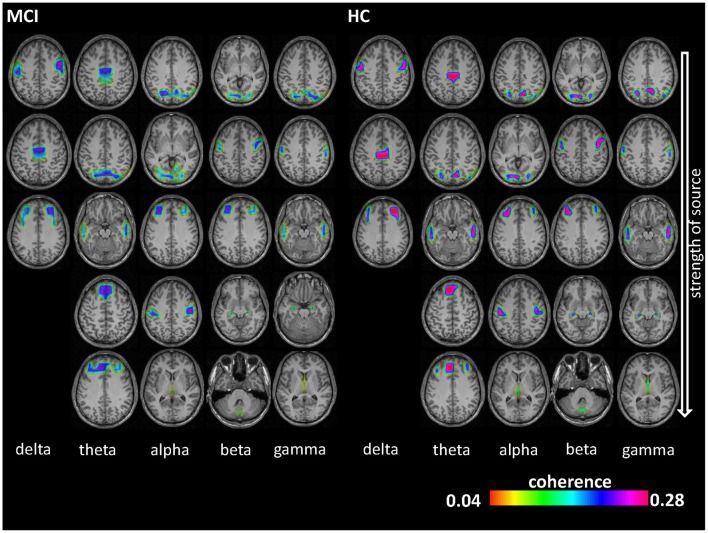
Electroencephalography (EEG) coherence sources results. Coherence sources are shown for the five examined frequency bands: delta (1–3 Hz), theta (4–7 Hz), alpha (8–13 Hz), beta (14–30 Hz) and gamma (30–49 Hz). Coherence can range from 0 to 1 (i.e., two sources have temporally fully aligned time courses). Sources are displayed on a template T1-weighted brain. The first source (1) in each frequency band is the highest power source and was later used as reference for determining the subsequent coherent sources.

As shown in Figure 1, the number of identified sources was lowest in the delta band (compared to the other frequency bands) and sources were present in Brodmann area (BA) 6 (premotor cortex), 32 (anterior cingulate cortex) and 9/46 (dorsolateral prefrontal cortex, DLPFC) in both groups. Theta-band related sources were located in BA 7 (precuneus), 9 (middle prefrontal gyrus), 21 (parahippocampal gyrus), 32, and 46. Different sources were involved in the alpha band, i.e., BA 4 (primary motor cortex), 17 (primary visual cortex), 23 (posterior cingulate cortex), 39 (angular gyrus), and 46. For beta frequency band, sources were spatially comparable between HC and MCI and were located in BA 6, 17, 34, 46 and cerebellum (label C). For gamma frequency band, sources were stronger in the HC group and comprised of BA 4, 21, 23, 34 and 39. Coherence between the sources was significantly different between the HC and MCI patients in the alpha (*p* = 0.009, *t* = 3.5) and beta (*p* = 0.01, *t* = 2.4) bands across all sources (Table 2A). Other frequency bands did not show any significant between-group differences.

**Table 2:** Global Electroencephalography (EEG) differences between controls and mild cognitive impairment (MCI).

	(A) Coherence—all sources (mean ± std)		(B) Source log power (mean ± std)		(C) RPDC (mean ± std)	
	MCI	HC	MCI	HC	MCI	HC
Delta	0.18 ± 0.03	0.21 ± 0.03	1.24 ± 0.03	1.27 ± 0.05	0.17 ± 0.03	0.18 ± 0.03
Theta	0.15 ± 0.02	0.18 ± 0.03	1.15 ± 0.06	1.15 ± 0.04	0.17 ± 0.04	0.16 ± 0.03
Alpha	0.14 ± 0.03*	0.19 ± 0.03	0.98 ± 0.07*	1.37 ± 0.06	0.15 ± 0.02*	0.21 ± 0.03
Beta	0.13 ± 0.03*	0.17 ± 0.02	0.89 ± 0.17	1.52 ± 0.09	0.19 ± 0.03*	0.25 ± 0.02
Gamma	0.09 ± 0.02	0.10 ± 0.02	0.83 ± 0.14	0.91 ± 0.14	0.13 ± 0.03	0.13 ± 0.02

std, standard deviation; RPDC, Renormalized Partial Directed Coherence RPDC. *p < 0.05, corrected.

### EEG: Power

The mean EEG source power from the first identified source for all five frequency bands were compared between the HC and MCI patients. Only in case of the alpha (*p* = 0.008, *t* = 3.7) and beta (*p* = 0.001, *t* = 4.7) frequency was there a significant absolute power difference between the two groups, with lower alpha and beta power in the MCI group (Table 2B). All the other frequency bands (i.e., delta, theta and gamma) showed no significant differences between the two groups.

### EEG: Directed Source Coherence (RPDC)

**Figure 2:**
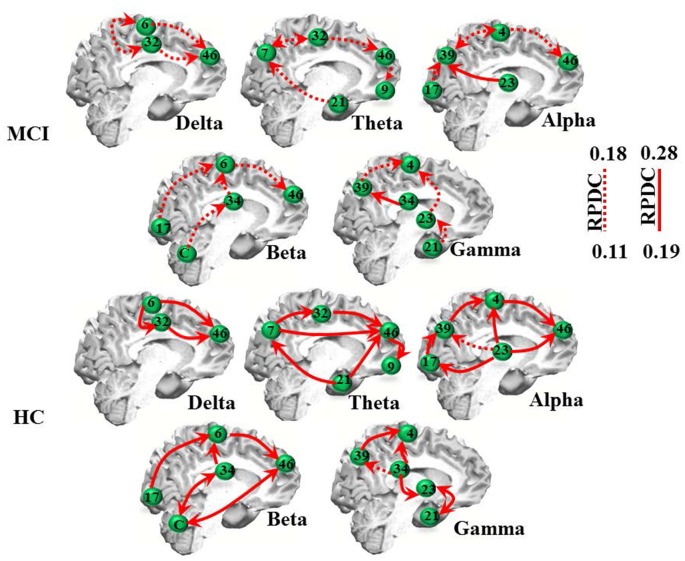
EEG renormalized partial directed coherence (RPDC) results. Top two rows: RPDC for mild cognitive impairment (MCI) participants. Bottom two rows: RPDC for healthy controls (HC). RPDC ranges between 0 and 1, and can be unidirectional or bidirectional (visualized by two red arrows). The labels indicate Brodmann areas (BAs).

The RPDC analysis revealed generally lower and more unidirectional connections in the MCI than in the HC group (Table 2C, Figure 2). As indicated in Figure 2, MCI patients showed weaker (RPDC maximum: 0.18) unidirectional connections in the theta band between frontal (BA 9/46, DLPFC) and parietal (BA 7, precuneus as part of the somatosensory association cortex), BA 32 and 21 (middle temporal gyrus) sources compared to the same unidirectional sources in HC (RPDC maximum: 0.28). For alpha, unidirectional connections were less strong between BA 4 (primary motor cortex), 46, 23, 17 and 39 in MCI. For beta, BA 6, 17, 34 (entorhinal cortex with parahippocampal gyrus), 46, and the cerebellum showed higher RPDC in HC than in MCI.

As summarized in Table 2C, the mean directed coherence (across all sources) for alpha (*p* = 0.002, *t* = 4.5) and beta (*p* = 0.002, *t* = 4.4) showed significant differences between the two groups. In contrast, delta, theta and gamma did not show any significant differences between the two groups.

We then tested, if the mean information flow between an identified source and all other sources was different between the two groups. A full list of group differences for each frequency band is provided in Table 3. For example, RPDC was stronger in the delta band in HC than MCI between all three identified sources. Specifically, the difference in the mean information flow of source 1 to source 2 and 3 was significant (*p* < 0.001, *t* = 12.97), i.e., weaker for MCI than HC. Additionally, the mean information flow from source 2 and 3 to source 1 was weaker in MCI than in HC than MCI (*p* < 0.00, *t* = −13.68). For the other frequency bands, we observed a similar pattern such as the (bidirectional) mean information flow was weaker in MCI than HC.

**Table 3:** EEG differences related to renormalized partial directed coherence (RPDC) between healthy controls (HC) and MCI.

Freq.	Source	Direction	*t*-value	*p*-value
delta	1	1	−12.97	*
	2	1	−13.62	**
	3	1	−8.01	**
	1	−1	−13.68	**
	2	−1	−11.65	**
	3	−1	−7.80	**
theta	1	1	−9.11	**
	2	1	−11.04	**
	3	1	−13.99	**
	4	1	−14.05	**
	5	1	−8.23	**
	1	−1	−14.09	**
	2	−1	−13.99	**
	3	−1	−10.59	**
	4	−1	−12.86	**
	5	−1	−8.12	**
alpha	1	1	−10.14	**
	2	1	−13.69	**
	3	1	−12.80	**
	4	1	−14.01	**
	5	1	−11.82	**
	1	−1	−7.36	*
	2	−1	−13.21	*
	3	−1	−13.82	*
	4	−1	−11.99	*
	5	−1	−12.56	*
beta	1	1	−12.45	*
	2	1	−9.92	*
	3	1	−11.82	*
	4	1	−8.33	*
	5	1	−12.18	**
	1	−1	−7.33	**
	2	−1	−9.09	**
	3	−1	−7.43	**
	4	−1	−7.80	**
	5	−1	−13.03	**
gamma	1	1	−12.10	**
	2	1	−9.38	**
	3	1	−13.94	*
	4	1	−7.35	*
	5	1	−10.26	*
	1	−1	−9.85	*
	2	−1	−12.61	**
	3	−1	−12.83	**
	4	−1	−8.45	**
	5	−1	−10.63	**

EC, eyes closed. A negative t-value indicates HC > MCI. *p < 0.001, **p < 0.0001.

### EEG—Clinical Interactions

In MCI, we observed an inverse correlation between global amyloid deposition and alpha coherence (*r* = −0.82, *p* < 0.001, *CI* = [−0.99 −0.43]) and RPDC (*r* = −0.56, *p* = 0.02, *CI* = [−0.91 0.02]) and gamma coherence (*r* = −0.68, *p* = 0.003, *CI* = [−0.88 −0.28]). These results indicate that the presence of ApoE4/E4 showed e.g., a lower alpha coherence than its absence.

MCI with a high genetic risk for AD (ε3/ε4, ε4/ε4) demonstrated lower alpha coherence and causal interactions than MCI with a normal genetic risk for AD (ε2/ε3, ε3/ε3). Specifically, the ApoE status was negatively associated with alpha coherence (*r* = −0.69, *p* = 0.002, *CI* = [−0.89 −0.38]), alpha RPDC (*r* = −0.74, *p* = 0.001, *CI* = [−0.91 −0.43]), and beta RPDC (*r* = −0.57, *p* = 0.02, *CI* = [−0.85 −0.13]). These results are highlighted in Figure 4. As the distribution of ε4 carriers vs. ε4 non-carriers was not uniform in our sample, we performed unpaired *t*-test analyses in addition to the reported correlation of ApoE and connectivity strength (Figure 4) for the following measures: alpha coherence, alpha RPDC, and beta RPDC. We compared a “no genetic risk group” (ε4 non-carriers, *n* = 25) to a “genetic risk group” (ε4 carriers, *n* = 9). We found that the connectivity strength was weaker for all these measures (alpha coherence: *p* = 0.001, alpha RPDC: *p* = 0.02, and beta RPDC: *p* = 0.009) in the “genetic risk group”, corroborating the results of the correlation analysis.

**Figure 3:**
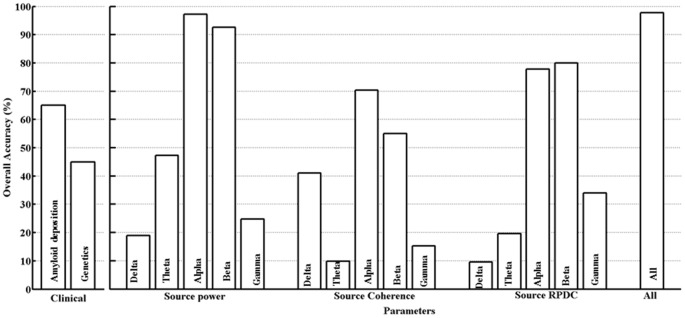
Support vector machine (SVM) classification results.

**Figure 4:**
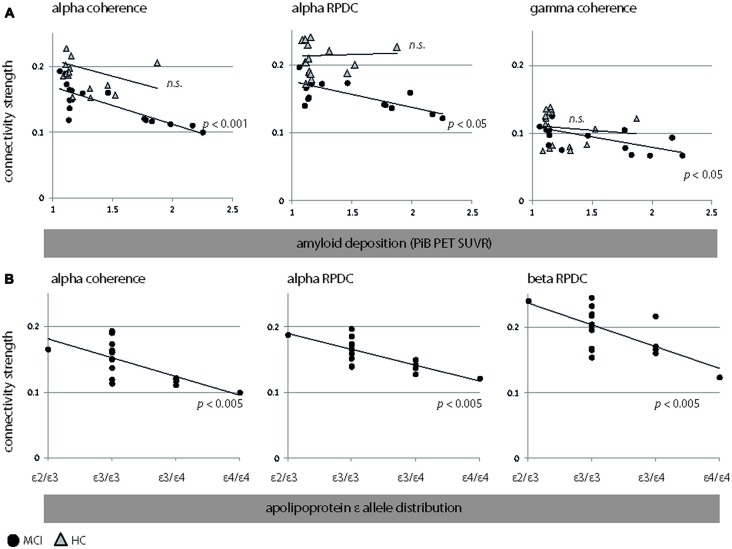
Significant associations between EEG connectivity measures (coherence and RPDC) and clinical parameters. **(A)** Interactions between EEG connectivity measures and amyloid deposition. We show results for HC (triangles) and MCI (filled circles). **(B)** Interactions between EEG connectivity measures and apolipoprotein allele (ApoE) occurrence (sorted by an increasing genetic risk for developing Alzheimer’s disease (AD): ε2/ε3, ε3/ε 3, ε3/ε4, and ε4/ε4) in MCI. Due to the non-normal distribution of amyloid beta (Aβ; *p* < 0.001, Kolmogorov-Smirnov test), and the ordinal character of ApoE (MCI: ε2/ε3, ε3/ε3, ε3/ε4, ε4/ε4), we applied nonparametric Spearman’s rank order correlation analysis. However, we did not perform this analysis for ApoE in HC, as ApoE was dichotomous (ε3/ε3, ε3/ε4). RPDC, renormalized partial directed coherence; SUVR, *standard uptake value ratio*.

A negative association was seen relating ApoE4-status and global amyloid deposition to alpha (*r* = 0.56, *p* = 0.01) and beta (*r* = 0.51, *p* = 0.02) source power in MCI. In HC, amyloid deposition was not linked to any EEG parameters (all *p* > 0.12). As for MCI, plots for alpha coherence, alpha RPDC, beta RPDC, and gamma coherence are shown Figure 4. Despite the fact that the groups were statistically homogeneous in age, we repeated EEG-PiB and EEG-ApoE analyses with age included as a nuisance variable (partial correlations). For MCI, alpha coherence (*r* = −0.8, *p* < 0.001, *CI* = [−0.96 −0.56]) and RPDC (*r* = −0.72, *p* < 0.001, *CI* = [−0.93 −0.33]), as well as gamma coherence (*r* = −0.63, *p* = 0.008, *CI* = [−0.86 −0.23]) were still significant after controlling for age. Additionally, beta RPDC showed a negative correlation to PiB (*r* = −0.55, *p* = 0.03, *CI* = [−0.83 0.09]). In the same group, ApoE showed comparable correlations to the above reported EEG parameters after correcting for age (alpha coherence: *r* = −0.68, *p* = 0.004, *CI* = [−0.87 −0.4]; alpha RPDC: *r* = −0.75, *p* = 0.001, *CI* = [−0.91 −0.43]; beta RPDC: *r* = −0.67, *p* = 0.005, *CI* = [−0.9 −0.02]). For HC, results remain unchanged, i.e., none of the EEG-PiB correlations were significant after controlling for age. Age was not correlated to any PiB, ApoE, or EEG parameters in any of the examined groups, except for an inverse correlation to beta power in MCI (*r* = −0.52, *p* = 0.03 *CI* = [−0.86 −0.04]). For the interaction between neuropsychological values, clinical values, and EEG we focused on CERAD recall, as this showed the strongest statistical difference between the HC and MCI groups (Table 1, *p* < 0.001, *t* = 4.7) relative to other neuropsychological variables. CERAD recall scores were not linked to PiB, ApoE, or EEG parameters in any of the examined groups (corrected or uncorrected for age).

We then tested for interaction effects between EEG parameters and PiB and ApoE across all particpants. We found signifcant interactions betweeen PiB and EEG power (all *p* < 0.001), coherence (all *p* < 0.001), and RPDC (all *p* < 0.001) in all examined frequency bands. For ApoE, interactions were seen with delta power (*p* < 0.001), delta coherence (*p* = 0.03), theta power (*p* < 0.001), theta RPDC (*p* = 0.02), alpha power (*p* < 0.001), alpha coherence (*p* = 0.001), alpha RPDC (*p* < 0.001). In addition, beta power (*p* < 0.001), beta coherence (*p* = 0.008), beta RPDC (*p* < 0.001), gamma power (*p* = 0.03), gamma coherece (*p* = 0.01), and gamma RPDC (*p* < 0.001) showerd a significant group by ApoE interaction.

### EEG—Cognitive Scores Interactions

Even after correction for multiple comparisons, we found some significant interactions, especially in the MCI group. In HC, alpha RPDC was inversely correlated to digit span forward (*p* = 0.009, *r* = −0.63) as well as to letter fluency scores (*p* = 0.02, *r* = −0.58). In contrast, coherence was positively correlated letter fluency scores (*p* = 0.04, *r* = 0.52). In MCI, delta RPDC was negatively associated VLMT recognition scores (*p* = 0.02, *r* = −0.62). For theta, MCI showed a negative association between coherence and VLMT word learning (*p* = 0.003, *r* = −0.73) and VLMT delayed recall scores (*p* < 0.001, *r* = −0.86). Alpha RPDC correlated positively with VLMT delayed recall scores (*p* = 0.008, *r* = 0.68). Beta power was negatively linked to Stroop task scores (*p* = 0.03, *r* = −0.57) and positively to VLMT learning (*p* = 0.009, *r* = 0.67) and VLMT delayed recall scores (*p* = 0.02, *r* = 0.63). Gamma power was positively associated with category fluency (*p* = 0.02, *r* = 0.63).

### SVM

The differentiation between the HC and MCI groups for all the clinical and estimated parameters in this study was assessed using a SVM classifier as described in the “Materials and Methods” Section. For the clinical parameters (PiB and ApoE), classification was 64.7% (PiB) and 45% (ApoE). With respect to EEG, only parameters (using delta-gamma power, coherence and RPDC as inputs) showing an overall classification accuracy above 75% will be discussed here (Figure 3). In case of the parameter alpha source EEG power, we were able to achieve accuracy of 97.3% (training), 87.1% (testing) and 96.2% (overall). The next parameter was alpha source directed connectivity; the achieved accuracy was 81.6% (training), 64% (testing) and 76.8% (overall). Using the beta frequency source EEG power as a parameter the accuracy was 95.2% (training), 77.3% (testing), and 93.6% (overall). The beta frequency directed connectivity values resulted in accuracy of 80.2% (training), 37.1% (testing) and 76% (overall). Finally, by including all the available parameters the accuracy was the highest with 99.6% (training), 87.4% (testing), and 97.8% (overall). Using age as covariate in the analysis did not significantly change the classification accuracies for the different clinical and EEG parameters (results not shown).

### Bayesian Power Analysis

To further validate the findings from the SVM analysis, we used Bayesian power estimation analyses, which provide complete distributions of credible values for group means and their differences (Kruschke, 2013). Specifically, we tested for EEG markers, with classification accuracy above 75%, for the capability of credible separation. The Bayesian analyses confirmed that for all included markers (alpha power, alpha RPDC, beta power and beta RPDC) group differences could be detected (effect size: 100%) with a CI of 95% using our sample size. In contrast, for theta power (classification accuracy below <40%), the capability of credible separation was only 61%.

## Discussion

In summary, MCI showed lower bidirectional information flow between fronto-temporal sources, including the hippocampus. Global amyloid deposition was negatively linked to alpha coherence and effective connectivity. Volunteers with a high genetic risk for AD demonstrated low alpha coherence and causal interactions. Classification accuracy for determining the cognitive state (HC or MCI) was high using EEG power, coherence, and RPDC as combined input parameters as well as for alpha source power on its own.

### Power

So far—and this is clearly a limitation compared to our study—other EEG studies just compared spectral power (on the electrode level) differences between MCI (or AD) and HC. Hence, it is difficult to compare our spatially more precise results on the source level to studies relying purely on scalp EEG power. For example, AD and MCI showed elevated delta and theta band power (Babiloni et al., 2007; Roh et al., 2011; Hatz et al., 2013) as well as EEG slowing (Dauwels et al., 2011). Furthermore, MCI and AD demonstrated reduced (relative) theta power at fronto-parietal and temporal electrodes (Claus et al., 1998; Jelic et al., 2000; Stomrud et al., 2010). Moreover, the study by Jelic et al. (2000) showed that alpha and theta power are good predictors to dissociate controls from MCI. In our study, we did not find altered theta source power between MCI and HC. This could have different reasons. First, as already explained, it is nearly impossible to compare source-related to scalp-related results. Any scalp related positive EEG result on theta power could simply be results of the influence of multiple sources that add up to elevated (or decreased) theta power. In addition, clinical factors might influence results on EEG power. In our sample, we carefully looked into the role of ApoE and PiB, which none of the existing studies did. Hence, we believe that the absence of theta power differences between MCI and HC are not contradictory to the literature but reflect rather state-of-the-art EEG post-processing and clinical assessment. Roh et al. (2011) demonstrated low alpha and beta power in AD. In line with this, our study revealed lower mean EEG alpha and beta source power in MCI relative to HC.

### Coherence

Some EEG studies have looked at coherence in MCI and AD (Jelic et al., 2000; Stam et al., 2003; Pijnenburg et al., 2004; Babiloni et al., 2009, 2016; Dauwels et al., 2009; Hsiao et al., 2013; Vecchio et al., 2014; Xu et al., 2014). For example, beta synchronization likelihood was decreased in AD (Stam et al., 2003). An EEG-based graph theory analysis and SVM was applied in MCI and early stage AD demonstrating accuracy of 93% discriminating MCI from controls (McBride et al., 2013). Recently, we reported shape alterations in subcortical regions in a similar MCI sample (Leh et al., 2016). We observed reduced inter-thalamic coherence too, which might be the result of the known (sub)-cortical structural impairments in MCI and AD (de Jong et al., 2011; Pievani et al., 2013; Cho et al., 2014). We found lower beta band related coherence in MCI in the entorhinal cortex, a region that is strongly affected by atrophy with increasing cognitive decline.

Prefrontal regions (i.e., BA 9 and 46) demonstrated lower delta-alpha coherence in the MCI group. The DLPFC is recognized for its role in working memory-related processes (Mars and Grol, 2007) and is functionally impaired in MCI and AD (Saykin et al., 1999). Furthermore, associative (BA 39), parietal (BA 7), and cingulate regions (BA 23), regions which are involved in attention processes, showed weaker coherence in MCI (Kondo et al., 2004). In addition, intra-regional coherence of the premotor and motor cortex (BA 4 and 6, respectively) was diminished across nearly all frequency bands in MCI. These changes extend an EEG study in early AD and MCI (Hsiao et al., 2014), which additionally reported altered connectivity in AD in parietal, cingulate, and medial regions, brain areas in which we also reported lower coherence in MCI. In general, volume conduction can especially impact undirected coherence, however by choosing the correct reference scheme like average reference (as used in this study) and the beamformer spatial transformation this effect can be avoided (Cohen, 2015a, b; Tenke and Kayser, 2015). In our study, gamma source coherence (and power) was stronger in HC than in MCI across all sources (although not significantly different between groups). This result was expected as gamma oscillations play a pivotal role in cognitive function. In mice, Colgin et al. (2009) demonstrated that fast and slow gamma information oscillations are relevant for information transfer and memory storage between areas of the entorhinal cortex. Moreover, the power of gamma oscillations positively correlates to working memory load in the prefrontal cortex. Alterations in gamma power (and coherence) have been reported in EEG studies examining patients with MCI and AD and it is known that altered gamma power is associated with hippocampal atrophy and memory impairment (Moretti et al., 2009a,b).

### Directed Coherence

A loss of long-range directional information flow has been reported in MCI and AD, reflected as lower alpha and beta parieto-to-frontal information flow (Vecchio and Babiloni, 2011). We found lower and overall less bidirectional information flow in MCI in these frequency bands as well as in theta. Yet, causal information flow in MCI was weaker from parietal-to-frontal sources, rather than the other way around, suggesting an imbalance in directed connectivity of a major attentional network. RPDC also identified abnormal information flow between cortical and sub-cortical regions, as beta RPDC was weaker between motoric and hippocampal areas in MCI. We found significantly stronger gamma band RPDC in HC compared to MCI for sources located in the entorhinal and mediotemporal cortex, which could indicate that even resting-state related gamma band oscillations are disturbed in MCI in brain regions associated with memory and attentional processing. Alterations in RPDC were also present in the angular and posterior cingulate cortex, areas that have been commonly linked to complex cognitive operations such as mental rotation or mental calculation (Nikolaev and Anokhin, 1997; Cebolla et al., 2014; Ueda et al., 2015). Lower gamma RPDC was additionally evident in MCI patients between sources of the parietal, entorhinal and primary motor cortex, indicating a disturbed connection between executive and attentional resources. We conclude that patients with MCI demonstrate impairments in gamma band related directed connectivity in brain regions traditionally associated with task-related processing.

We found lower RPDC in the cerebellum in MCI than HC. Cerebellar degenerative processes can lead to deficits in verbal and nonverbal intelligence, verbal associative learning, and visuospatial skills (Akshoomoff et al., 1992). Regarding undirected connectivity, it is known that frontal coherence in the alpha band is linked to fiber tracts of the cerebellum and other brain regions in MCI and HC (Teipel et al., 2009). Altered functional and effective EEG connectivity among long-range cortical networks (i.e., fronto-parietal and fronto-temporal) has been reported in MCI and AD in the frequency range <12 Hz (Babiloni et al., 2004; Teipel et al., 2016). Additionally, Koenig et al. (2005) also reported in two independent data sets reduced coherence in MCI and AD in the alpha, beta and gamma bands. Directed connectivity changes (using EEG) of the cerebellum have not been examined in the context of MCI or AD. We observed lower beta RPDC of the cerebellum to the DLPFC (BA 46) and entorhinal cortex (BA 34, parahippocampal gyrus) in MCI compared to HC. We suggest that the decreased directed connectivity in the cerebellum in MCI might reflect a sign of disturbed connectivity to important brain regions involved in long-term memory (such as the entorhinal cortex) as well as executive control and attention (such as the DLPFC).

### The Link between Amyloid Deposition and ApoE to EEG Measures

Current AD models revealed that the cortical EEG is linked to the presence of Aβ (Wang et al., 2002; Bobkova et al., 2008; Jyoti et al., 2010; Corbett et al., 2013; Schneider et al., 2014). For example, lower theta and elevated beta/gamma activity was found in transgenic mice carrying mutated amyloid precursor protein (APP), which mimic certain features of AD and which develop Aβ a few months after birth (Wang et al., 2002). In middle-aged rats, it could be shown that the recombinant form of secreted APP elevated low frequency cortical and hippocampal EEG power, indicating the effect of Aβ on neuronal function (Sánchez-Alavez et al., 2007). In a computational model, it was recently found that Aβ can induce (hippocampal) theta band power changes (Zou et al., 2011). A recent study examined in patients with early stages of AD the link between Aβ42 (based on measures from the cerebrospinal fluid), phosphorylated tau protein (p-tau), and (resting-state) EEG alpha dipolarity and its standard deviation (Kouzuki et al., 2013). The authors found a negative correlation between alpha dipolarity and p-tau as well as between alpha dipolarity and Aβ42/p-tau. Our study highlights the link between abnormally high levels of cortical amyloid and altered EEG measures in MCI, reflected by lower alpha coherence and RPDC. We hence suggest that significant amyloid burden affects global neuronal network properties as we found effects of cortical amyloid burden on both undirected and directed EEG coherence. No interactions were seen in HC between cortical amyloid and any EEG parameter, indicating that only an elevated level of amyloid burden (as seen in MCI) leads to reduced inter-regional connectivity.

An increased genetic risk for developing AD was associated with reduced undirected (alpha) connectivity and (alpha and beta) directed connectivity. This suggests that ε4 allele presence reduces neuronal communication in EEG power rhythms required for guiding important cognitive-attentional and executive functions (Klimesch, 2012). Especially because of the triple association observed between coherence (and RPDC), amyloid deposition, and ApoE ε4 status, one can ask if the changes in those EEG bands are the consequence of elevated amyloid deposition (Babiloni et al., 2013). Alternatively, this might be due to an increased genetic risk for developing or elevated p-tau protein levels (Ferrazzoli et al., 2013). Answering this question is beyond the scope of the present study, as this would require the examination of cortical levels of tau or large enough cohorts (including HC and MCI) with and without elevated amyloid and ε4 allele presence. Nevertheless, our results are in line with EEG studies that reported an association between ApoE and (source) power, specifically those that found a more pronounced EEG slowing in ApoE ε4 positive participants (Lehtovirta et al., 1996, 2000; Ponomareva et al., 2008). The observed interaction between alpha power and ApoE seems to be already present in young (−21 years) ε4 carriers (Lee et al., 2012). Furthermore, these subjects showed reduced functional connectivity not only in our study (i.e., as negative interaction between the ApoE status with alpha mean coherence, alpha mean RPDC, beta mean RPDC, and gamma mean source coherence), but also in another qEEG study (Jelic et al., 1997).

### SVM

SVM has been used recently to classify AD and MCI patients with functional connectivity from functional magnetic resonance imaging (fMRI) data, achieving a 75% accuracy distinguishing healthy participants from MCI, and 97% accuracy distinguishing MCI from AD (Challis et al., 2015). A new hyper-network brain connectivity method was used for classification of MCI from HC using fMRI (Jie et al., 2014). Diffusion tensor imaging data have been used previously for classification using SVM for MCI (Dyrba et al., 2015), MCI and AD (Jung et al., 2015), and MRI-based classification between AD and MCI (Yang et al., 2013). Also here, good classification accuracy was achieved (86.9%) for example differentiating MCI from AD (Jung et al., 2015). EEG resting state source based coherence between AD and MCI has been shown earlier to have differences in delta coherence in the sensorimotor network (Hsiao et al., 2014). The importance of beta oscillations in differentiating MCI from HC have been previously discussed during cognitive performance tasks (Güntekin et al., 2013). In another study using beta frequency from EEG as an integrative biomarker for predicting the disease progression from MCI to AD (Poil et al., 2013). Nevertheless, all the above-mentioned studies have used fMRI for the classification in SVM or in EEG at selective frequency bands and tasks. In this study, we have used a more global whole-brain approach to search for the ideal biomarkers (i.e., frequency band specific EEG power, coherence, effective connectivity) to differentiate between HC and MCI. We found a higher overall classification accuracy (97.8%). Ultimately, the alpha source power showed the highest parameter classification accuracy, which highlights the essential role of alpha oscillations during resting state for differentiating between the two cognitive states. Using a logistic regression model, both delta power and ApoE can separate patients with very early stages of probable AD from MCI (Hatz et al., 2013). Furthermore, the classification was even better (trend) for the combined compared to the EEG only model. However, this effect was not seen in our combined model (which contained EEG power from the delta to the gamma band) and might rely on the fact that we have only studied patients with MCI (relative to HC) and not patients with AD. Another reason for the weak classification results using ApoE might be that we do not have many participants with an ApoE ε4/ε4 configuration. We think that the classification (using ApoE or PiB) is lower compared to other studies as we aimed to distinguish not MCI due to AD, which is characterized by positive amyloid—PET from HC, but MCI as a heterogeneous entity defined by cognitive impairment from HC (i.e., healthy aging).

### Limitations

Due to the small group sizes, our study might be considered exploratory. Importantly, however, we argue that the observed findings (on coherence and RDPC) do represent reliable group differences according to the results of the Bayesian power analyses. This important statistical justification (for our sample size and the robustness of our results) is often not performed in other EEG studies on MCI (or AD) with similar or even larger sample sizes. In addition, all results were corrected for multiple comparisons using Bonferroni correction in order to minimize false positive results. Furthermore, this is one of the few studies which considers EEG, amyloid burden, and genetic risk in both MCI and HC. We cannot exclude the presence of vascular damage as we did not record T2 or FLAIR images. Yet, we can argue than none of our subjects had any significant stenosis (based on the evaluation of the MR angiography images).

## Conclusion

This study found that both functional and directed connectivities are sensitive to cognitive alterations, amyloid deposition and genetic risk, as the distribution and directionality of functional connections differ between brains of “healthy” elderly and participants with MCI.

## Author Contributions

LM, FR and AFG recorded the data. LM, MM and ARA analyzed the data. FR, PGU, SK, SEL, MS and CH critically reviewed the article.

## Conflict of Interest Statement

The authors declare that the research was conducted in the absence of any commercial or financial relationships that could be construed as a potential conflict of interest.

## Footnotes

^1^http://doc.pmod.com/pneuro/7674.htm

^2^http://www.clidyn.ethz.ch/arfit/
